# The Role of Therapeutic Anticoagulation in COVID-19

**DOI:** 10.1155/2020/8835627

**Published:** 2020-08-27

**Authors:** Ruth McGovern, Patrick Conway, Isabell Pekrul, Omar Tujjar

**Affiliations:** ^1^Department of Anaesthesia, Intensive Care and Pain Medicine, Sligo University Hospital, Sligo, Ireland; ^2^Department of Anaesthesia, Ludwig-Maximilians-University of Munich, Munich, Germany; ^3^Department of Transfusion Medicine, Cell Therapeutics and Haemostasis, Ludwig-Maximilians-University of Munich, Munich, Germany

## Abstract

Coagulopathy has proven to be a common complication of the novel coronavirus SARS-CoV-2, with evidence of elevated D-dimers and fibrin degradation products associated with an increased incidence of thromboembolism. Despite emerging evidence describing the coagulopathy and its clinical relevance in COVID-19, fewer studies have addressed the potential role of empiric therapeutic anticoagulation in this setting. We report the case of a patient admitted to our intensive care unit (ICU) with severe acute respiratory distress syndrome (ARDS) secondary to COVID-19 whose clinical trajectory improved dramatically after initiation of a therapeutic dose of LMWH. The patient showed progressive elevation of fibrinogen and D-dimers despite a prophylactic dose of LMWH during her ICU stay. This was met with a moderate increase of troponin T-hs, an escalating need for vasopressors, and a progressive decrease in her P/F ratio despite preserved lung static compliance. Her platelet count was normal and had an elevated fibrinogen during the first week of ICU stay. The ECG was normal, and a bedside transthoracic echocardiogram showed no evidence of pulmonary embolism and a preserved EF with no regional wall motion abnormalities (RMWA). The chest X-ray was not dissimilar to previous exams, and the ABG showed hypoxia with normal pCO_2_ values. The decision was made to commence empiric therapeutic enoxaparin. The patient did not experience bleeding complications, and her clinical trajectory appeared to change dramatically. She was successfully extubated three days later and proceeded to clinical recovery and eventual discharge from the ICU. The available evidence shows that there is undoubtedly coagulopathy associated with COVID-19 with various subsequent forms of clinical manifestation described in the literature. Evidence also shows the benefits of heparin as an anticoagulant. From the discussion of this case report, however, it can be concluded that despite the plausible theoretical rationale, studies pertaining to the role of empiric therapeutic anticoagulation in this setting fall short of providing compelling evidence. Subsequently the role of empiric therapeutic anticoagulation in COVID-19 remains unclear with a pressing call for further research.

## 1. Introduction

Coagulopathy has proven to be a common complication of the novel coronavirus SARS-CoV-2 [1]. Despite emerging evidence delineating the nature of the coagulopathy and its clinical relevance in COVID-19, much fewer studies have addressed the potential role of empiric therapeutic anticoagulation in this setting. We present the case of a patient admitted to our intensive care unit (ICU) with severe ARDS secondary to SARS-CoV-2. The patient manifested a dramatic clinical improvement after initiation of empiric therapeutic anticoagulation.

## 2. Case Presentation

A 74-year-old female who presented with shortness of breath, increased work of breathing, and fever was admitted to the ICU in Sligo University Hospital, Ireland. She had a background of stage 2 smoking-related chronic obstructive pulmonary disease (COPD). Her cardiovascular history was unremarkable. She reported feeling generally unwell for approximately 7 days before presenting to the emergency department where she was found to have an SpO_2_ of 68% on room air.

A working diagnosis of community-acquired pneumonia was made based on X-ray findings, elevated inflammatory markers, and the history provided. On auscultation, fine crepitations and wheeze were heard throughout. With a notion of high likelihood of COVID-19, she was promptly transferred to the ICU for emergency intubation and stabilisation. A polymerase chain reaction (PCR) test was run on a nasopharyngeal sample which tested positive for SARS-CoV-2.

The patient subsequently developed severe acute respiratory distress syndrome (ARDS). She received mechanical ventilation as per current standards of care. Inflammatory biomarkers were continuously monitored. D-dimers, ferritin, C-reactive protein, triglycerides, and lactate dehydrogenase were all found to be elevated. Among other medications, the patient was receiving a prophylactic dose of enoxaparin (40 mg SC OD).

**Figure 1:**
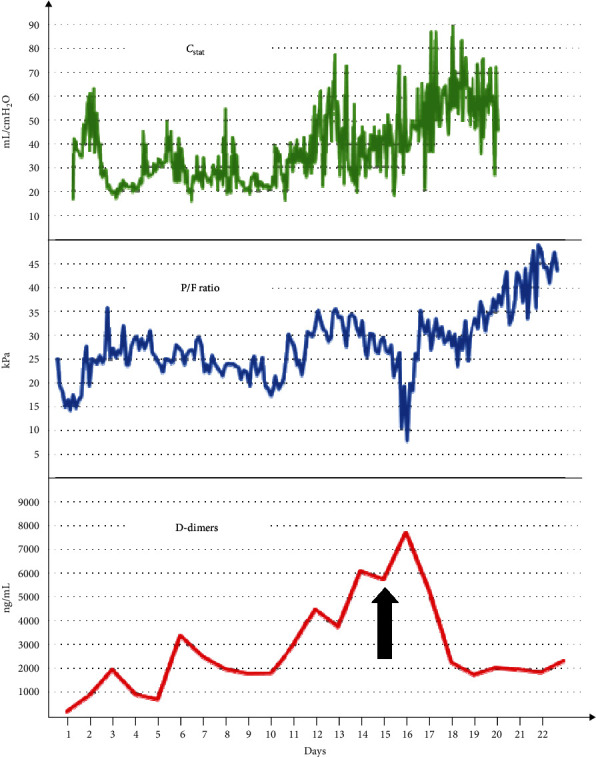
Trend comparison between D-dimers, P/F ratios, and lung static compliance. Arrow: commencement of empiric therapeutic anticoagulation.

Fourteen days into her ICU stay, there was evidence of gradual elevation of D-dimers (peak 7655 ng/mL). This was met with a moderate increase of troponin T-hs (peak 115 ng/L), an escalating need for vasopressors (peak 0.6 mcg/kg/min), and a progressive decrease in her P/F ratio (nadir 8.37 kPa) despite preserved lung static compliance (48.2 ± 15.3 mL/cmH_2_O during the deterioration period) (Figure 1). Her platelet count was normal and had an elevated fibrinogen (peak 680 mg/L) during the first week of her ICU stay. The ECG was normal, and a bedside transthoracic echocardiogram showed no evidence of pulmonary embolism and a preserved EF with no regional wall motion abnormalities (RMWA). Compressive ultrasonography showed no evidence of DVT in the lower limbs. The chest X-ray was not dissimilar to previous exams, and the ABG showed hypoxia with normal pCO_2_ values.

The decision was made to commence empiric therapeutic enoxaparin (1 mg/kg SC BD). Considering the impossibility to conduct further cardiac diagnostics in that context, empiric dual antiplatelet therapy was also commenced following advice from cardiology (ASA 300 mg and ticagrelor 180 mg STAT followed by ASA 75 mg OD and ticagrelor 90 mg BD). The patient did not experience bleeding complications, and her clinical trajectory appeared to change dramatically. She was successfully extubated three days later and proceeded to clinical recovery and eventual discharge to a medical ward after spending 24 days in the ICU. The patient expressed her consent to publish this anonymised case report.

## 3. Discussion

Several recent studies carried out in quick succession have reported coagulopathy to be a common complication of the novel coronavirus SARS-CoV-2. Research suggests that severe COVID-19 displays evidence of vascular dysfunction, thrombosis, and dysregulated inflammation [1]. Studies describe elevated D-dimers and fibrin degradation products (FDP), mild thrombocytopaenia, and prolonged prothrombin time [2] with the occurrence of disseminated intravascular coagulation (DIC) [3], higher rates of venous thromboembolism (VTE), and cerebrovascular accidents in critically ill COVID-19 patients [4] as well as central venous line and extracorporeal circuit thrombosis [5]. The question as to whether or otherwise there is a role for empiric therapeutic anticoagulation in the setting of coronavirus, however, remains unclear. This is in part due to the relative infancy of this current pandemic and as a result the subsequent paucity of knowledge and thus meaningful research on the subject. This review is aimed at analysing recent pertinent literature and academic debate that forms current theories in relation to the role of empiric therapeutic anticoagulation in COVID-19.

The nature of the coagulopathy seen in COVID-19 has been repeatedly characterised by elevated D-dimers and fibrin degradation products (FDP), mild thrombocytopaenia, and prolonged prothrombin time with pulmonary coagulation and fibrinolysis purported to be influenced by, and correlate to, certain proinflammatory cytokines [2, 6–8]. Viral injury, abnormal release of cytokines, and damage-associated molecular patterns (DAMPs) are thought to induce localized microvascular inflammation. Subsequently, there is activation of endothelial cells leading to vasodilation and prothrombotic conditions [1]. DIC also appears to play a role. DIC is a well-known complication of sepsis which may include COVID-19-related sepsis. It occurs following tissue injury whereby cytokines are released secondary to monocyte and endothelial cell activation with the expression of tissue factor and release of von Willebrand factor. Free thrombin can activate platelets with subsequent acceleration of systemic coagulation, which is poorly controlled by circulating endogenous inhibitors [9]. At the late stages of COVID-19, it appears that fibrin-related marker levels, i.e., D-dimer and FDP, are elevated in all deaths. This may indicate a common coagulation activation and secondary hyperfibrinolysis condition in these patients [3].

The discussion pertaining to coagulopathy in COVID-19 was heralded by Tang et al. [3]. This was following a number of studies describing the clinical and biochemical characteristics of patients infected with COVID-19. Guan et al. were among the first to observe raised D-dimer levels with higher values seen in severe cases and those requiring mechanical ventilation or suffering various other outcomes including death [2]. Such evidence was similarly illustrated in other studies [6–8]. Tang et al. produced two retrospective observational studies. Results of the first found that coagulopathy especially elevated D-dimer and fibrin degradation products and longer prothrombin time featured more in nonsurvivors than survivors [3]. They also reported that more nonsurvivors fulfilled the criteria for DIC when compared with survivors, 71.4% vs. 0.6%, respectively. Further work was a larger retrospective observational study of 449 patients with severe COVID-19 [6]. The study found that there was no difference in 28-day mortality between heparin and nonheparin users. This, however, was lower in patients with a sepsis-induced coagulopathy (SIC) score > 4 or a D-dimer result > 6-fold the upper limit of normal. In summary Tang et al. have illustrated the presence of significant coagulopathy in nonsurviving patients when compared to survivors with more of the former satisfying criteria for DIC. It is further suggested that anticoagulant therapy reduces mortality in such patients. To this end, this would suggest that there is a role for empiric therapeutic anticoagulation in cases of severe COVID-19.

Nevertheless, there are several limitations related to this study that are worth noting. Firstly, it is a retrospective study. Secondly, the authors provide that 94 patients were administered enoxaparin 40-60 mg/day. Not only did they not clarify the proportion of patients that received which dose, but they also did not delineate the route of administration. This obscurity is further compounded by use of the word “therapy” which may be denoted as all patients being therapeutically anticoagulated [10]. This, however, is not in keeping with the doses outlined above. Moreover, in terms of patients fulfilling criteria for SIC of >4, it would appear that it was falsely augmented as the SIC score used was doubly weighted in error and thus may lead to inaccurate interpretation of the study [6], not to mention that DIC is in and of itself associated with high mortality rates independent of the sepsis that may or may not be associated with COVID-19. For these reasons, the work by Tang et al. may prove misleading insofar as no patient appears to have received therapeutic anticoagulation, and the benefit of prophylactic anticoagulation is long established.

Clinical relevance of the coagulopathy seen in COVID-19 is evident in the literature. Acute pulmonary emboli (PE) have been described in a small number of case reports [11, 12]. Klok et al. in a study of 184 ICU patients with COVID-19 found a compound 31% incidence of venous and arterial thromboembolism in patients receiving at least standard doses of thromboprophylaxis [13]. In their prospective cohort study, Helms et al. reported an incidence of thrombotic complications in 42% of 150 COVID-19 patients with ARDS despite prophylactic or therapeutic anticoagulation [5]. Both studies would also suggest that there may be a role for empiric therapeutic anticoagulation in the setting of COVID-19. The high incidence of PE in critically ill patients is an important consideration. The clinical presentation of PE has historically been variable. It is for this reason that diagnosis is frequently missed and it is often diagnosed during autopsy of critically ill patients. Notwithstanding the differences in patient cohorts, a retrospective study by Girardi et al. [14] illustrated an incidence of PE of 30.4% in a population of critically ill patients, which is at odds and higher than the PE incidence reported by Helms et al. The latter also employ the use of historical non-COVID ARDS patients as controls [5]. Historical controls naturally introduce an element of noncontemporaneous control bias [15] as they may not be subjected to the same in-depth examination and investigation as a result of PE not being a suspected diagnosis. This is especially the case when historical controls are compared to the ongoing scrutiny of current COVID-19 patients during this global pandemic in an effort to gather information and best formulate meaningful medical management. While the work by Helms et al. is highly informative, the above considerations illustrate potential methodological pitfalls.

Other studies have described new-onset cerebrovascular disease (CVD) in COVID-19 patients [4]. Here, 13 of 221 patients developed CVD following infection, 11 of which were acute ischemic strokes. The CVD patient cohort was older and had cardiovascular and cerebrovascular risk factors. The authors suggest that such findings indicate that older COVID-19 patients may be more likely to develop CVD which may require closer attention. They also hypothesize that the increase in inflammatory response may be the cause of coagulopathy and subsequent CVD. While this study highlights the occurrence of acute cerebral ischemia in the setting of COVID-19, it remains a single-centre retrospective study with much weight attributed to patient-specific variables that are known risk factors for CVD in the absence of COVID-19.

With emerging research, endothelial disease is increasingly hypothesized to be a contributing disease process in COVID-19 [16]. Pulmonary shunting has been associated with intense vasodilation and endothelial dysfunction [1, 17] with reports illustrating increased respiratory dead space perhaps secondary to lung-vascular thrombosis from thrombotic microangiopathy or pulmonary embolism [17, 18]. The coagulation system, however, is an inherent and anticipated part of the immune response [19]. Many inflammatory diseases, such as various vasculitis, result in thrombosis. To illustrate, DVT in Behcet's disease, and other forms of vasculitis, result from inflammation of the vessel wall as opposed to the existence of a hypercoagulable state. Moreover, anticoagulants have proven to provide no benefit in these settings [20, 21]. The coagulation system is also triggered by the presence of hypoxia which is a well-known cause of hypercoagulability in many disease states such as chronic obstructive pulmonary disease (COPD) and obstructive sleep apnoea syndrome (OSAS) [22, 23]. Hypoxia is known to induce platelet aggregation and activation of blood coagulation [24]. This is of significance because hypoxia is a cardinal feature of COVID-19. Coagulation is further triggered by macrophages who play a central role in fibrinolysis. They form a part of the pathophysiology of COVID-19 by way of secondary haemophagocytic lymphohistiocytosis (sHLH) [25].

To this end, while there is no uncertainty that D-dimers are elevated, such a phenomenon is not unique to COVID-19. D-dimer elevation may be attributable to inflammation, hypoxia, bleeding, and macrophages as part of the physiological immune response. It has very low specificity in the critically ill population as a result of the many disease processes that are associated with fibrin turnover [14]. While D-dimer elevation has been purported to be a poor prognostic value, Yin et al. demonstrated no significant difference in D-dimer level between 449 COVID-19 patients and 104 non-COVID patients, all with severe pneumonia [7, 8].

In terms of potential treatment options, much discussion has centred around heparin as a promising contender. Tang et al. purport a reduced mortality for heparin treatment in COVID-19 cases [5, 6]. The shortcomings of this study as outlined above, however, call into question the validity of these conclusions and may be considered to be somewhat misleading. Heparin has also been discussed favourably by Thachil [26]. This piece outlines the bidirectional relationship between the immune system and thrombin production whereby the inflammatory response may be attenuated by the action of heparin inhibiting thrombin. They also outline heparin's innate ability to bind to inflammatory cytokines, disabling neutrophil chemotaxis, inhibiting the complement factor C5a, and sequestering acute-phase proteins. Undoubtedly, heparin has a beneficial role in terms of certain forms of inflammation [27]. In the context of inflammation secondary to other disease processes such as vasculitis, it has not shown to be of benefit [20, 21]. Whether it would assist in the coagulopathy secondary to COVID-19, however, is unknown. Another important consideration in relation to antithrombotic therapies is the occurrence of drug interactions with antivirals used in COVID-19 in terms of their active metabolites and competition for certain CYP450 enzymes. Ineffective doses of clopidogrel and enhanced effects of ticagrelor have been seen when used with lopinavir/ritonavir [28, 29]. Rivaroxaban and edoxaban also are contraindicated with lopinavir/ritonavir for similar reasons [30]. Heparin may be the most suitable option in terms of anticoagulation in COVID-19 with other agents not approved for use in the critically ill or still under scientific examination.

At this time of global pandemic, with its inherent uncertainty, the International Society on Thrombosis and Hemostasis (ISTH) has released prompt interim guidance on the recognition and management of coagulopathy in COVID-19. They propose low molecular weight heparin (LMWH) as a management strategy for COVID-19-associated coagulopathy. They purport that LMWH should be considered in all patients with COVID-19 requiring hospital admission, including the noncritically ill, in the absence of contraindications. Moreover, they suggest that patients with markedly elevated D-dimers, prolonged prothrombin time, platelet count < 100 × 10^9^/L, and fibrinogen < 2.0 g/L receiving prophylactic LMWH that are disimproving should receive blood products based on whether they are “bleeding” or “nonbleeding” [31].

Although timely and informative, these guidelines prove challenging for a number of reasons [32]. Firstly, the idea of making clinical decisions, such as admissions, discharges, and escalation to critical care, based on chemical biomarkers such as D-dimer, prothrombin time, and platelets is somewhat concerning. It is arguably safer and more appropriate to base such decisions on the patients' entire clinical picture taking into account their comorbidities, clinical trajectory to date, and the presence or absence of associated complications which may impact prognostication. To base significant decisions on coagulation biomarkers is to assume that D-dimers are the product of secondary fibrinolysis alone. Given that it is uncertain in the setting of COVID-19, as to whether the origin of D-dimers is primary or secondary fibrinolysis, such an assumption may be unwise. Akima et al. are also arguably justified in expressing disconcertion at the recommendation for blood product administration to patients with coagulopathy in the absence of bleeding. This is at odds with widely accepted transfusion guidelines [33] and has not shown to improve outcomes in the setting of DIC.

The recommendations set out by the ISTH have also been discussed by Barrett et al. who propose the use of unfractionated heparin for systemic anticoagulation as opposed to LMWH [34]. It is long established that significantly high levels of fibrinogen result in heparin resistance [35, 36]. Moreover, it is not uncommon for COVID-19 patients to have fibrinogen levels > 700 mg/dL with some reaching 900 mg/dL which is uncommon in the ICU outside that of COVID-19 [34]. This would suggest that LMWH or unfractionated heparin prophylactic doses may be ineffective in severe COVID-19. Unfractionated heparin may act to ensure therapeutic levels with ongoing monitoring of coagulopathy, although this could prove to be difficult considering the FXII deficiencies seen with COVID-19 [37]. It is for these reasons that Barrett et al. call for a more aggressive regimen for systemic anticoagulation than LMWH.

Another important consideration is the treatment of the fibrin deposits already laid down in the alveolar spaces as part of the pathophysiology of COVID-19. A recent proposition by Moore et al. is the use of tissue plasminogen activator (tPA) in the treatment of acute respiratory distress syndrome ARDS [38]. Discussion pertaining to tPA is further explored in a case series by Wang et al. involving the unlicensed use of alteplase in COVID-19 patients with ARDS and associated respiratory failure [39]. Results yielded an initial improvement in the P/F ratio, varying between 38% and 100%, in all 3 cases. Improvements in the P/F ratio displayed a transient nature insofar as they were lost over time after the completion of tPA. It is possible that longer lasting improvements may be demonstrated by redosing patients that demonstrate transient improvement with tPA [40]. It can be ascertained that while anticoagulation may have a role in terms of prevention of fibrin deposition, both locally and systemically, its role may prove futile in the setting of already formed fibrin deposits for which tPA may be beneficial.

This brings the discussion to whether or otherwise these patients should be therapeutically anticoagulated at all. The evidence shows that there is undoubtedly coagulopathy associated with COVID-19 notwithstanding that coagulopathy goes hand in hand with inflammation. Well-established evidence also shows the benefits of heparin as an anticoagulant in various settings including its favourable influence on the inflammatory process. Despite the plausible theoretical rationale, however, the current evidence fails to show any benefit of empiric therapeutic anticoagulation in this setting. While it may be reasonable to assume that therapeutic anticoagulation would only benefit a disease process characterised by hypercoagulability, published studies outlined above unfortunately fall short of meaningful evidence. Subsequently, due to the lack of compelling evidence, clinicians are practicing a variation of anticoagulation regimes. While it is possible and reasonably probable that there is a role for empiric therapeutic anticoagulation in critically ill COVID-19 patients with severe ARDS characterised by increasing D-dimers and deteriorating oxygenation with preserved lung compliance, there is certainly a call for further research. Moreover, if benefit is confirmed, further investigation ought to address anticoagulant agents of choice and optimal dosing. At this time of uncertainty and infancy, the publishing of retrospective informative research, desperately sought after by medical centres worldwide, must be weighed up with the necessity for more powerful and compelling evidence that may be provided by more extensive prospective studies.
